# Climate Change Impacts and Workforce Development Needs in Federal Region X: A Qualitative Study of Occupational Health and Safety Professionals’ Perceptions

**DOI:** 10.3390/ijerph18041513

**Published:** 2021-02-05

**Authors:** Katherine M. Pedersen, Tania M. Busch Isaksen, Marissa G. Baker, Noah Seixas, Nicole A. Errett

**Affiliations:** 1 Department of Environmental and Occupational Health Sciences, School of Public Health, University of Washington, Seattle, WA 98105, USA; kate.pedersen131@gmail.com (K.M.P); tania@uw.edu (T.M.B.I.); bakermg@uw.edu (M.G.B.); nseixas@uw.edu (N.S.); 2 Department of Health Services, School of Public Health, University of Washington, Seattle, WA 98195, USA; 3 Department of Urban Design and Planning, College of Built Environments, University of Washington, Seattle, WA 98195, USA

**Keywords:** occupational health and safety, climate change, climate-related hazards, Pacific Northwest, education, training, key informant interviews

## Abstract

Climate change is considered one of the top health threats in the United States. This research sought to (1) to understand the perceptions of occupational health and safety (OHS) professionals regarding the impacts of climate-related hazards on OHS in Region X, and (2) to explore the ideas of these OHS professionals regarding the content of future training programs that would better prepare OHS professionals to identify and mitigate climate-related hazards in Region X. Key informant (KI) interviews with 17 OHS professionals familiar with the climate-related hazards and impacts to OHS in Region X were coded and thematically analyzed. Climate hazards, social and economic impacts from climate-related hazards, and sector-specific worker and workplace impacts from climate-related hazards were described as having interacting relationships that influenced worker health and safety impacts. KIs further described how workplace controls could be used to mitigate OHS impacts of climate-related hazards, and how training of the OHS workforce could influence the ability to successfully implement such controls. Our findings suggest that OHS impacts are sector-specific, influenced by social and economic factors, and can be mitigated through workplace controls designed and implemented by a trained OHS workforce. The findings from this work should inform future educational and training programming and additional research and translation activities in the region, while our approach can inform other regions as they develop regionally specific OHS climate change training and programming.

## 1. Introduction

Climate change is now recognized as one of the leading health threats of the 21st century [1] and will alter existing occupation-related hazards and exposures and create the opportunity for new risks [2,3]. Specific climate-related hazards that affect occupational health and safety (OHS) include increases in ambient temperature and air pollution, UV radiation, extreme weather, changes to vector-borne disease range [3]. In addition, transitions to emerging industries, changes to the built environment, mental health impacts, economic impacts, as well as potential new hazards due to advances in geoengineering have the potential to influence OHS [4]. Given regional variation in the impacts of a changing climate, the resultant OHS issues are likely to be regionally specific. Yet, the regional variation of these impacts has yet to be comprehensively explored to inform geographically tailored OHS responses, including through targeted training of the OHS workforce.

Federal Region X, which consists of the U.S. states of Alaska, Idaho, Oregon, and Washington, is on the front-lines of climate change. The region has warmed considerably over the last 100 years [5]; in fact, Alaska is warming faster than any other U.S. state [6]. Across the region, the impacts of this changing climate are already being experienced or anticipated. For example, wildfires and subsequent smoke events are a growing annual hazard both in prevalence and magnitude [7,8,9,10], exposing workers, particularly those who work outside, to harmful levels of air pollution. Additionally, climate change is also expected to broaden the geographical range of many vector borne diseases, putting workers, especially those who work outdoors, at increased risk [2,11,12,13,14,15]. Cascading impacts to work and workplaces highlight the need for more broad reaching research on the implications of climate-related hazards to OHS within the region.

The large and growing number of outdoor workers in Federal Region X makes climate-related OHS impacts of particular interest. Utilizing updated Region X state employment data and projections as reported by Doubleday et al. (2019) [16], there were approximately 7.1 million employed persons in Federal Region X in 2018 (data from 2019 for Oregon). About 408,000 of these workers are in construction and extraction occupations, and 167,000 are in farming, fishing, and forestry occupations, which include work that is traditionally performed outdoors. It is estimated employment in these outdoor occupations will grow by almost 8% in the region over the next 10 years, similar to the employment growth in the region as a whole. Notably, this is likely an underestimate since these cross-sectional counts and projections do not include undocumented, part-time, contingent, self-employed, migrant, and temporary workers. 

Yet, with the exception of OHS impacts associated with increased ambient temperatures [12,17,18,19,20,21,22,23], relatively little research has been done to understand climate change’s impacts to workers in the Federal Region X. This work has largely focused on agricultural workers, and has not explored broader impacts, determinants of, or solutions to climate-related OHS risks in the region. There is a growing need for systems-level research that identifies key OHS issues of concern and possible solutions in the context of the region’s climate impacts. In response, this exploratory research sought to (1) to understand the perceptions of OHS professionals regarding the impacts of climate-related hazards on OHS in Region X, and (2) to explore the ideas of these OHS professionals regarding the content of future training programs that would better prepare OHS professionals to identify and mitigate climate-related hazards in Region X. The findings from this research have the potential to inform graduate and continuing educational programming and inform additional research and translation activities in the region, while serving as a model approach for developing regionally specific OHS climate change training and programming across the U.S. 

## 2. Materials and Methods 

**Approach:** We conducted and thematically analyzed semi-structured key informant interviews with OHS professionals in U.S. Federal Region X. 

**Participant Recruitment:** We recruited key informants (KIs) to participate in semi-structured interviews using a combination of purposive and snowball sampling. Purposive, or purposeful, sampling is a non-probability sampling technique common to qualitative research to recruit participants who have a high likelihood of being able to provide rich information to elucidate a particular concept or phenomenon [24]. Our sampling approach sought to identify individuals who met the following inclusion criteria: “occupational health and safety professionals familiar with the emergent and evolving hazards associated with climate change in Federal Region X and associated OHS risks.” Given our focus on identifying responsive graduate and continuing education opportunities, as a secondary consideration, we sought to identify individuals familiar with the Northwest Center for Occupational Safety and Health (NWCOHS), a National Institute for Occupational Safety and Health (NIOSH)-funded Education and Research Center (ERC) based at the University of Washington, its programs, and OHS professional responsibilities of NWCOHS trainees and alumni. While not all participants were familiar with the NWCOHS, all those recruited had professional proficiency in OHS. Our purposive sampling approach attempted to include KIs from a range of professional roles, occupational foci, relationships with the NWCOHS (e.g., trainee, employer of trainee(s), etc.) and years of OHS experience. We continued to identify KIs through snowball sampling, where we invited additional participants recommended by other participants that were determined to meet inclusion criteria, until thematic saturation of meta themes was attained.

We leveraged the extensive professional network of the NWCOHS to identify prospective informants. The NWCOHS is the only NIOSH-funded training center in Federal Region X. As such, many OHS professionals in the region have trained in our formal graduate or continuing education programs or have hired or worked with our trainees. 

**Data collection:** Semi-structured interviews were conducted by using an interview guide developed a priori. Interviews were conducted by a single member of the research team (KMP) over the phone between May and July 2019. 

Interview questions are provided in Supplemental Materials. Due to concerns from prospective KIs regarding their employer and the use of the terminology “climate change”, the interview guide was altered after four interviews to use the terminology “emerging or evolving climate-related hazards”. However, the substance of the interview guide did not change. Interviews were recorded and professionally transcribed. KIs provided verbal informed consent prior to interview commencement and recording. 

**Data analysis:** A combined deductive and inductive approach was used to code the transcribed interview textual data. In the deductive phase, codes were developed to reflect the key domains explicitly explored through the interview guide, as well as broad categories of climate-related OHS impacts previously identified in the literature [3,4]. In the inductive phase, transcripts were then read and re-read to identify additional categories, or themes, that emerged. These broad categories were formalized into codes with specific definitions and instructions on when they should be applied. 

Codes were applied using NVivo Qualitative Analysis Software. In an effort to refine the codebook, two members of the research team (KMP and NAE) independently coded 12% (*n* = 2) of the interview transcripts and compared results. Discrepancies in code application were adjudicated via consensus building discussion and the codebook was updated accordingly. A single member of the research team (KMP) coded the remainder of the transcripts.

Interview contact sheets were developed for each interview, summarizing the themes that were present in that interview. Contact sheets were shared with the KIs, allowing them the opportunity to review and “member check” (i.e., participant validation) the interviewer’s interpretation of the key themes for accuracy and completeness [25]. Analytic memos were then developed by code to summarize key themes that emerged across the interviews. Finally, matrices and tables were developed to explore themes within, and across interviews [25]. 

**Ethics:** This research was determined to be human subjects research that qualifies for exempt status (category 2) by the University of Washington Human Subjects Division.

## 3. Results

A total of 51 individuals were contacted by email and invited to participate in the study. Seventeen individuals agreed to participate in the interviews. KIs were employed by a variety of sectors, including academia, government, and industry, and had between five and 36 years of experience (Table 1). KIs worked in, or represented, Washington, Oregon, and Alaska, or were federal OHS employees with familiarity of the region. One KI was a resident of British Columbia, Canada.

Our analysis explores key climate-related hazards, OHS impacts, workplace controls, social and economic impacts, worker and workplace impacts, and climate-related training for OHS professionals. Each theme is summarized in Table 2 and discussed individually below.

### 3.1. Climate-Related Hazards

KIs discussed hazards that are likely to change in frequency or magnitude in the region because of climate change, which may have implications for OHS. Several climate-related hazards, both ongoing and emerging in the region, were identified by KIs as having impacts or potential impacts to OHS. Rising temperatures, wildfires and wildfire smoke, and extreme weather events were all identified by the majority of KIs as a concern for Region X workers, either now or in the future. Flooding and coastal erosion, the spread of vector range and/or pathogens, and drought were also mentioned by a few KIs. In addition, the following climate-related hazards were mentioned as potentially impactful to work or OHS by a small number of KIs: sea-level rise, cold events, wind, air pollution not related to wildfire smoke, ocean acidification, UV radiation, and increased pollen levels. However, climate change denial was indicated by several KIs as a challenge to addressing the impacts of climate-related hazards to OHS in Region X.

### 3.2. Work and Workplace Impacts

KIs described experienced or prospective impacts on the type of work, amount of work, manner of work, and/or people who will be performing work in Federal Region X. Nearly all KIs discussed some type of climate impact to the type or quantity of work performed in Region X, as well as to workplaces themselves. The two most common concerns were changes to how work is currently being done—such as changes to daily tasks—and supply and demand impacts—such as increasing or decreasing workloads. Furthermore, a few KIs mentioned evolving technologies in automation are impacting OHS negatively in the region. In addition, a couple of KIs indicated that sustainability practices (e.g., waste reduction), implemented in response to climate change are influencing workplaces by encouraging eco-friendly practices in the work environment. Another KI also pointed out that there is a lack of research on OHS related to work performed in emerging green industries. 

The majority of KIs discussed supply and demand issues within the workforce due to regional climate-related hazards. Many KIs indicated demands for particular types of work would increase in response to hazards caused by climate change. This was explained by one KI as the need for more construction, both due to changing green industries and damage caused by hazards, would increase the impacts of construction to progressing climate change; “*The biggest challenge that I see is that in order to mitigate climate change, you have to do a lot of construction, but construction is also the biggest source of CO2 emissions out there. So, it contributes a huge amount to the problem that’s leading to climate change*.”

### 3.3. Occupational Health and Safety (OHS) Impacts

Nearly all KIs identified experienced or prospective OHS impacts from regional climate-related hazards in the region. Many KIs indicated heat-related illness, pathogen/vector-related illness, and mental health as climate-related OHS impacts. Examples of heat-related health outcomes mentioned by KIs included: traumatic injuries, heat stroke/stress, cardiovascular disease, dehydration, and death. Additionally, one or two KIs indicated the following OHS impacts due to emerging or evolving climate-related hazards: respiratory illness, slips, trips, and falls, toxic chemical exposure during green industry construction, and exhaustion. One KI mentioned that workers could potentially experience a decrease in everyday exposures due to a reduction in work time resulting from an inability to work during climate-related extreme events.

At-risk populations identified included guest workers (both documented and undocumented), indigenous populations, and volunteer groups. KIs indicated that this vulnerability was due to immigration status, a lack of familiarity to the area or language, poor or non-existent training, or being part of a population known to be vulnerable in the community. As one KI mentioned: “*[M]arginalized workers who might not have legal immigration status who are most likely to be intimidated, bullied, threatened by their employers*.” 

Outdoor workers, including those working in the agriculture, emergency response, construction, commercial fishing, logging, and public works sectors were identified as being at elevated risk to emerging and evolving climate-related hazards in Region X. KIs discussed the inability for outdoor workers to move inside when conditions could impact health and safety (e.g., during periods of increased temperature, poor air quality, during wildfires, or other extreme weather events).

Several KIs also identified indoor workers and drivers as populations that are at-risk, but who are likely to be forgotten or ignored during climate-related events. These KIs stressed the importance of protecting indoor populations due to the overall lack of air conditioning in Region X and because rising temperatures will impact working conditions within buildings. Drivers, or those who spend most of their working hours in a vehicle, were identified as an at-risk group by several KIs. KIs pointed out that drivers are often alone while working with little available support if they were to encounter an emergency. Additionally, these KIs described how drivers are often left out of OHS discussions. 

### 3.4. Social and Economic Impacts

Social and economic factors that impact a worker’s ability to mitigate or adapt to emerging or evolving climate-related hazards were identified. Some KIs mentioned that not all workers have the same access to OHS controls, or capacity to adapt and respond to emerging climate-related hazards. One KI discussed that conditions present at home and work, such as lack of air conditioning during hot nights, can impact workers’ ability to adapt, especially to rising temperatures. Other KIs indicated that not all workers were in a financial position to be able to risk losing wages or their jobs due to missing work because of OHS concerns or illness caused by a climate-related event. “*[T]hat’s always my number one answer, at the top of the list, is that workers are—many, many workers who are living paycheck to paycheck are the ultimate vulnerable population. And all of their families are vulnerable because of that, as well. And so, I know that a lot of workers don’t feel empowered to speak up and protect their own safety and health*.”

A few KIs described climate-related socioeconomic impacts, such as those related to income and industry, as having cascading impacts to wellbeing. For example, one KI from a federal agency discussed how changing temperatures could result in seasonal delays and yield reduction for aquaculture and agriculture industries, resulting in both economic and mental health consequences for workers. 

### 3.5. Workplace Controls

KIs identified administrative controls, such as changing how, when, and where work is being done, and personal protective equipment (PPE) as specific workplace controls which can protect workers from climate-related hazards. They described workplace controls that were currently in place, as well as those that would be needed in the future. 

The majority of KIs discussed changes to how, when, and where work is being done due to emerging regional climate-related hazards. For example, one KI mentioned that there will need to be changes to when work is performed, with the possibility that outdoor tasks may need to be performed at different times of day. However, many KIs discussed that there is an overall reluctance to change working practices due to climate-related hazards, and one KI indicated that change would be a very slow process. Prioritization of economic productivity over health and safety was mentioned by a few KIs as a reason employers are reluctant to change. For example, one academic KI discussed the Dungeness crab industry and how the lack of quotas incentivizes workers to work no matter the risk. Because of this, this KI stressed that changing weather patterns could have dire OHS consequences if fisheries continue to incentivize risky work environments. 

Other administrative controls at the employer-level largely focused on providing workers with the necessary information to perform work safely in the face of climate-related hazards. A few KIs discussed needs to improve or augment communication practices, especially with the emergency response and agriculture workforces, including about emergency planning. Requiring administrative controls through regulation was identified as an area that needed more work by a few KIs. 

Changes to regulatory safety nets were mentioned by a few KIs as impacting OHS. One KI expressed concern that OHS standards, such as those enforced by OSHA or an OSHA-approved state plan, have decreased over time and are insufficient to protect workers from emerging or evolving climate-related hazards in the region Additionally, many KIs indicated that there was a lack of regulatory support for stricter and adaptive OHS measures in the workplace. For example, one KI from the federal sector mentioned OSHA standards do not apply to disaster volunteers and indicated that there is no oversight that guarantees a safe workplace for these individuals. Furthermore, current legislation and the political environment were described as a challenge to the OHS environment. For example, one KI stated that they did not believe there would be much regulatory activity in increasing OHS standards, especially with additional workplace controls, under the then current U.S. presidential administration (2019).

Several KIs described PPE as a potential way to protect workers from climate-related hazards. Most of these KIs noted that PPE design or its use will need to evolve along with climate-related hazards. One KI discussed that emerging or evolving climate-related hazards will force workers to use PPE they may be unfamiliar with, presenting challenges.

### 3.6. Climate-Related Training for OHS Professionals

Specific knowledge, skills, and/or abilities were mentioned by all KIs as something OHS professionals will need when adapting to emerging and evolving climate-related hazards in the workplace. Additionally, some KIs mentioned specific training that should be integrated into OHS educational programs going forward. 

A few KIs discussed the lack of a one-size-fits-all solution as an impediment to training OHS professionals about workplace controls for climate-related hazards. For example, one KI said there was no “*lift with your legs, not with your back*” solution to worker protection in the face of new and emerging risks. A few KIs mentioned that a better collaboration between researchers and industry leaders and decision-makers, and their inclusion in the development of research questions, could help address this barrier. 

#### 3.6.1. Knowledge

Nearly all KIs identified some type of knowledge requirement for OHS professionals, both in the context of graduate training and continuing education, around emerging and evolving regional climate-related hazards. Many KIs described the need to integrate emerging or evolving risks into more OHS competencies. Additionally, many KIs discussed the need to communicate job-related risks to workers, with a few specifying emergency responders as a primary population of concern. 

Several KIs also discussed the need for climate literacy among workers and employers. A few KIs discussed specific industries, such as logging and commercial fishing, that they perceived as having not fully recognized that the climate is changing. They indicated that this lack of recognition created challenges related to changing to OHS practices moving forward. These KIs discussed how educating these industries about evolving hazards and their impact on industry could promote OHS practice changes to better protect workers.

#### 3.6.2. Abilities and Skills

Several KIs mentioned specific skills and abilities that OHS professionals will need to protect workers in the face of emerging or evolving climate-related hazards. Many KIs mentioned that OHS professionals need communication skills to better communicate the evolving risks to industries and workers, and to empower workers to advocate for better OHS controls (e.g., through effective interactions with their elected representatives). As one KI put it, “*There’s not a prescriptive solution to this crisis, but there is a political solution to this crisis and that political solution depends on community, labor, political organizing, and so I think that the workers in civil society that [are] affected by these health risks from climate change [are] not going to be able to organize, have the capacity to do that, if we don’t increase our leadership development of communication skills and organizational skills within civil society organizations*.”

A few KIs discussed the need for applied learning for OHS trainees to gain a better understanding of the OHS profession outside of academia. They suggested hands-on training, role play, first aid training and internships as ways for OHS students to apply their classroom knowledge to the ‘real world’. One KI said, “*I would basically tell a graduate student, ‘If you are interested in policy research, you should spend some time at the [state] State Legislature to sort of see how completely creative it is, and how science sometimes has minimal effect on creating public policy.’ So, I would really ask for those types of applied experiences that would be helpful in sort of cultivating future [OHS professionals] to be effective. Because just writing up a public health master’s thesis around some data analysis just [isn’t] going to cut it*.”

Finally, a few KIs mentioned evolving climate-related hazard and risk identification trainings, including to identify health risks and symptoms, would be important going forward. Additionally, others described ideas for hosting regular training on regional climate-related topics. One KI explained how the availability of new web-based training allows for rapid and just-in-time training on evolving hazards. They indicated that existing trainings could be adapted to incorporate climate-related content. 

## 4. Discussion 

In the face of climate change, this timely study aimed (1) to understand the perceptions of OHS professionals regarding the impacts of climate-related hazards on OHS in Region X, and (2) to explore the ideas of these OHS professionals regarding the content of future training programs that would better prepare OHS professionals to identify and mitigate climate-related hazards in Region X. Our findings suggest that climate-related hazards are already being experienced in the region, that their OHS impacts are sector-specific, influenced by social and economic factors, and that there are ongoing needs and opportunities to mitigate these impacts through workplace controls designed and implemented by a trained OHS workforce. KIs identified a number of gaps within the existing literature relevant to Federal Region X workers including: health and safety impacts to indoor and driver populations due to climate-related hazards, mental health impacts of climate-related hazards, the state role in addressing regional-specific workplace hazards associated with climate change, and how the education of future OHS professionals will need to evolve in order to address these concerns. While recognizing these appreciable gaps in evidence, our results indicate there is an immediate opportunity to improve climate literacy, communication skills, and risk assessment skills for the OHS workforce.

Our findings suggest that climate-related hazards experienced among workers in the region include: rising ambient temperatures, wildfires and wildfire smoke, extreme weather events, flooding and coastal erosion, the spread of vectors and/or pathogens, and drought. While the existing literature has described the impacts of heat on agriculture workers, both at home and on the job [12,19,20,21,22,26,27], wildfires on both firefighters and workers who are inundated with wildfire smoke for days [28,29,30,31], and how extreme weather will impact both workers and the infrastructure they interact with [3], our findings indicate that additional research is needed to better understand and address how OHS professionals can better prepare employers and workers for a changing climate with emerging and evolving hazards.

Our findings also suggest that the existing body of evidence on climate-related OHS impacts does not fully address impacts or populations of concern to regional OHS professionals. For instance, previous research has focused on the immense impact climate-related hazards have on outdoor workers [2,3,4,11,12,13,17,18,21,22,23,32,33]. However, KIs discussed how indoor workers are at risk for adverse health and safety outcomes when faced with climate-related hazards. For instance, due to the low prevalence of air-conditioned spaces in many areas in the region, KIs discussed increased risks to indoor workers associated with rising ambient temperatures. Given costs that employers will face in order to retrofit existing workplaces, this is likely to be a continued challenge.

In addition, drivers who spend the majority of their working hours operating a vehicle (e.g., truckers, ride-share, taxi, etc.), were identified by KIs as an at-risk occupational cohort and have been largely absent from the existent literature on climate-related OHS impacts. Drivers were discussed by KIs as being particularly high-risk because they are often alone with little support, which is particularly concerning in an emergency situation. According to one study by the U.S. Department of Transportation, Washington and Oregon had above average risk of weather-related commercial motor vehicle crashes in 2005–2006, with Oregon having the highest risk rating. Alaska and Idaho were just below average risk [34]. Ride-share drivers are another driver population of particular concern due to the increasing popularity of rideshare apps like Lyft and Uber. As climate change progresses, risks to these working populations may increase without proper protections. The need to identify and implement sustainable, effective solutions to reduce risk may become increasingly urgent.

Our findings suggest that marginalized populations may be at increased risk of OHS impacts of climate change. These workers, whose social and economic precarity place them at increased risk of experiencing health consequences of exposure, may also lack the power or self-efficacy to avoid exposure or demand appropriate workplace controls. For example, migrant farm workers may be unable to miss work during high heat days, or when wildfire smoke rolls in, because they cannot afford to miss a day of wages. Moreover, fear of losing their jobs altogether may impede their willingness to ask for changes to work hours or access to PPE to reduce heat or smoke exposure, respectively. Additional research is necessary to understand the disproportionate impacts of climate change on low-wage and precarious workers, as well as the cascading consequences on health and wellbeing. Such information can inform policy and regulatory activities to minimize exposure and protect their health, safety, and social and economic wellbeing.

Mental health impacts were of particular concern to KIs and have been described previously in the literature as an impact of climate change on workers and among the general public [3,13,35,36]. As workers are firstly members of their communities, our findings reinforce the need for public health interventions to address mental health impacts of climate change, given their potential to exacerbate OHS impacts. The 2020 COVID-19 pandemic has highlighted the impacts that both OHS and employment have on worker mental health, especially for front-line medical workers [36]. This provides an opportunity to explore the ways in which rapidly changing work environments, which may also result from climate-related events (e.g., transitioning to working from home, decreased staffing, and changing OHS guidance and/or standards) impact worker mental health.

KIs also suggested opportunities to integrate climate change into ongoing graduate and professional education of the OHS workforce. For instance, they suggested general climate literacy education, integration of climate change-related examples and case studies into curricula, training on climate change communication, and training on climate change-related risk assessment as ways to better prepare future OHS professionals. Due to the diversity of impacts expected and the breadth of workers exposed, customizable, hazard-specific training modules or case studies may be an effective approach to introducing climate hazards into mainstream OHS curricula. KIs stated that curricula should be hands-on or built using real-world examples. They suggested this would better prepare graduate students for the challenges they will encounter while trying to address the OHS impacts of climate change in the region. This, coupled with improved communication skills that bridge the gap between research, industry, and policy sectors, is an opportunity to improve climate literacy in fields which have done little to adapt to ongoing changes in the climate. NIOSH—funded ERCs can lead the way in developing regionally-specific OHS modules and case studies that integrate local hazards and resonate with at-risk worker groups in their service area.

Finally, the need for additional controls, including those required by OHS regulation, to improve OHS in the face of emerging and increasing climate-related hazards was identified. As all states in Federal Region X, except Idaho, at least partially regulate OHS through OSHA-approved State Plans [37], there is significant opportunity in the region to address regionally specific climate hazards through state-level OHS regulation, as well as assess their effectiveness. As California and Washington are currently the only states that have attempted to address heat-related illness concerns through state-level OHS regulations and heat illness controls [18], there is precedent for such policy innovation in the region. Trained, forward thinking OHS professionals have the potential to improve existing regulations that are inadequate to protect workers against the existing and emerging hazards associated with climate change, especially the most vulnerable populations [38].

Limitations: As our study aimed to identify Federal Region X-specific climate-related OHS impacts and opportunities for OHS professional training and education, the findings presented herein may not be generalizable beyond the region. However, we submit this as a model approach for use across the U.S. to identify regional climate-related OHS professional concerns and responsive education and training programs. Moreover, while our KIs were those with expertise in OHS, we did not interview front-line workers who may have different perspectives regarding the hazards they face and their impacts. Not all KIs invited to participate did, specifically those within the labor and union industries, and those who did not participate may differ systematically from those who did. Finally, given the politicization of climate change in the United States, it is possible that KIs may have tempered their responses because of employer policies or fear of retribution. 

## 5. Conclusions

Emerging and evolving climate-related hazards are already impacting workers in Federal Region X. Heat, wildfires, and extreme weather hazards are of particular concern, with cascading impacts to the types, timing and location of work performed, the physical and mental health and of workers, and social and economic conditions of workers. Administrative controls, regulations, and PPE offer opportunities to reduce risk. Additional skills-based training in applied settings can improve the OHS workforce’s preparedness to deal with emerging climate-related hazards in the workplace. Additional research is needed to assess the impacts of climate-related hazards on specific under-studied populations and sectors, like indoor workers and drivers, the mental health impacts of climate-related hazards, the effectiveness of state-level regulation for climate-related OHS, and the impact of climate-related educational programs for OHS professionals.

## Figures and Tables

**Table 1:** Key informant professional demographics.

Sector Demographics		
Sector	Total	Average Years of OHS Experience
Academic	5	15
Federal	5	36
State	2	12
Local	2	14
Private	1	30
Non-Profit	1	5
Union/Labor	1	20
Total	17	19
**Geographical Demographics**		
**Location**	**Total**	
Washington	12	
Oregon	1	
Alaska	2	
Federal	1	
British Columbia	1	
**Familiarity with NIOSH or NWCOHS**		
**Program**	**Total**	
NIOSH	11	
NWCOHS	14	

**Table 2:** Key Themes Summary with Specific Examples.

Theme	Summary	Examples
Climate—related hazards	Many hazards were identified as emerging or expanding climate-related OHS risks in Region X: Rising temperatures, wildfires and wildfire smoke, extreme weather events, flooding and coastal erosion, the spread of vector range and/or pathogens, wind, air pollution not related to wildfire smoke, ocean acidification, UV radiation, and increased pollen levels. Rising temperatures was the most commonly mentioned climate-related hazard by KIs, followed by wildfires and wildfire smoke, and extreme weather events	Rising temperatures put indoor workers at increased risk due to lack of air conditioning in the region Wildfires are impacting rural and urban workers due to the wide geographical spread of wildfire smoke Workers in coastal Alaska operate heavy machinery in storm swells to help prevent flooding
Work and workplace impacts	KIs discussed how there would be changes to the type or quantity of work being done, such as changes to daily tasks and changes to workloads Emerging technologies were discussed as a work and workplace impact by a few KIs	Increasing risk to workers, infrastructure, and heavy equipment, such as excavators and barges, due to extreme weather events Indoor construction work in extreme heat and/or with poor ventilation Decreasing health and safety culture
Occupational health and safety impacts	OHS impacts mentioned by KIs included heat-related illness, pathogen/vector-related illness, mental health, respiratory illness, slips, trips, and falls, and exhaustion Mental health impacts of climate-related hazards were identified as climate-related health outcomes that have not been sufficiently researched KIs identified worker populations at increased risk to OHS impacts, including outdoor workers (agriculture, emergency response, construction, commercial fishing, logging, and public works sectors), indoor workers, and drivers	Death due to “idle free” company policy (causing high heat in vehicles) Exhaustion due to decreased recovery time between wildfire seasons
Social and economic impacts	KIs described climate-related socioeconomic impacts, such as those related to income and industry, as having cascading impacts to wellbeing	Decreasing employment need in recreational and tourism industries due to low snow and water levels Increases in migration and changing demographics due to sea level rise Decreasing health and safety standards due to potential economic and employment losses
Workplace controls	Administrative controls, such as how, where, and when work is done, provision of OHS information, changes in regulatory safety nets, and PPE were the most commonly discussed workplace controls KIs discussed that not all workers have the same access to OHS controls, or capacity to adapt and respond to emerging climate-related hazards	Outdoor workers possibly having to perform tasks at different times of day
Climate-related training for OHS Professionals	All KIs acknowledged that OHS professionals will need specific knowledge, skills, and abilities in order to address emerging and evolving climate-related hazards in the workplace KIs mentioned specific climate-related training that should be integrated into OHS educational programs going forward	OHS professional trainings mentioned included applied learning opportunities such as role play, first aid training and internships, and regionally specific climate-related hazard and risk identification

## Data Availability

The data presented in this study are available on request from the corresponding author. The data are not publicly available due to concerns related to participant confidentiality.

## Supplementary Materials

The following are available online at https://www.mdpi.com/1660-4601/18/4/1513/s1, Interview Questions.
