# Supplementary material for: Climate Change Impacts and Workforce Development Needs in Federal Region X: A Qualitative Study of Occupational Health and Safety Professionals’ Perceptions
Source: Int J Environ Res Public Health. 2021 Feb 5;18(4):1513. doi: 10.3390/ijerph18041513 (PMC7915234; doi:10.3390/ijerph18041513)
Supplement: Supplementary file 1 [file ijerph-18-01513-s001.pdf]

Interview Questions

1. To start out, we would like to hear about your current professional role. Can you tell me a little about what you do as it relates to occupational safety and health?
   Prompts:
   - *Can describe the industry you currently work in/regulate?*
   - *How long have you worked in this industry?*
   - *What about any past experience in other industries?*

2. Are you familiar with the University of Washington's National Institute of Occupational Safety and Health (NIOSH)-funded Education and Research Center (ERC), also known as the Northwest Center for Occupational Health & Safety?
   - *If so:*
     - *How long have you been associated with or known about the ERC?*
     - *In what capacity have you been involved with the ERC? Student? Alumni? Faculty? Employer of ERC grads? NIOSH employee?*
     - *What relationship do you have with the ERC in your current role?*
   - *If not:*
     - *Are you familiar with the National Institute for Occupational Safety & Health*
     - *(Interviewer explain what NIOSH is, what the ERC is, etc)*

3. How will emergent or evolving climate-related hazards influence the health and safety risks faced in workplaces in the Pacific Northwest
   Prompts:
   - *How will changes in frequency, intensity or duration of extreme heat wildfire smoke events, or disasters affect occupational health and safety? How about vector borne diseases?*
   - *What types of occupational exposures and health/safety risks are associated with these types of events?*
   - *Which workers are most at risk? Why?*
   - *How will workplace controls change?*

4. What new health risks will workers face due to emerging or evolving climate-related hazards?

5. What knowledge, skills, and/or abilities will occupational health professionals need to be able to protect workers from evolving or increasing occupational risks associated with emerging or evolving climate-related hazards?

6. What sorts of continuing education courses do occupational health professionals need with regards to the health impacts of emerging or evolving climate-related hazards?
   Prompt: For example, through coursework, experiential activities, other requirements?

7. Is there anyone else you'd suggest we talk to about this topic?

8. Those are all of the questions we have for you today. Is there anything else you'd like to tell us?

9. Do you have any questions for us?
